# Supplementary material for: The soluble glutathione transferase superfamily: role of Mu class in triclabendazole sulphoxide challenge in Fasciola hepatica
Source: Parasitol Res. 2021 Jan 27;120(3):979–91. doi: 10.1007/s00436-021-07055-5 (PMC7889535; doi:10.1007/s00436-021-07055-5)
Supplement: Supplementary file 1 — (PDF 127 kb) [file 436_2021_7055_MOESM1_ESM.pdf]

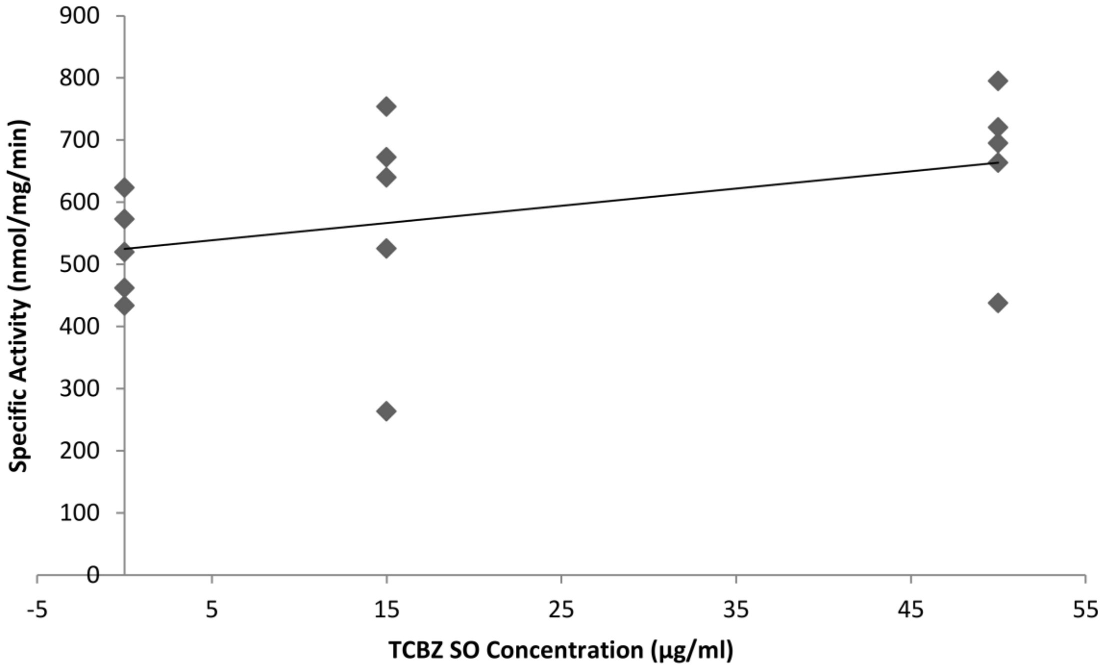

Online Resource 1 Glutathione transferase specific activity (nmol/mg/min) assessed with the model substrate CDNB for *F. hepatica* cytosolic samples for each TCBZ-SO treatment. Treatment groups, control, sub-lethal, and lethal, contained adult fluke that were exposed to TCBZ-SO at 0, 15 and 50 µg/ml respectively
