# Supplementary material for: The soluble glutathione transferase superfamily: role of Mu class in triclabendazole sulphoxide challenge in Fasciola hepatica
Source: Parasitol Res. 2021 Jan 27;120(3):979–91. doi: 10.1007/s00436-021-07055-5 (PMC7889535; doi:10.1007/s00436-021-07055-5)
Supplement: Supplementary file 3 — (PDF 127 kb) [file 436_2021_7055_MOESM3_ESM.pdf]

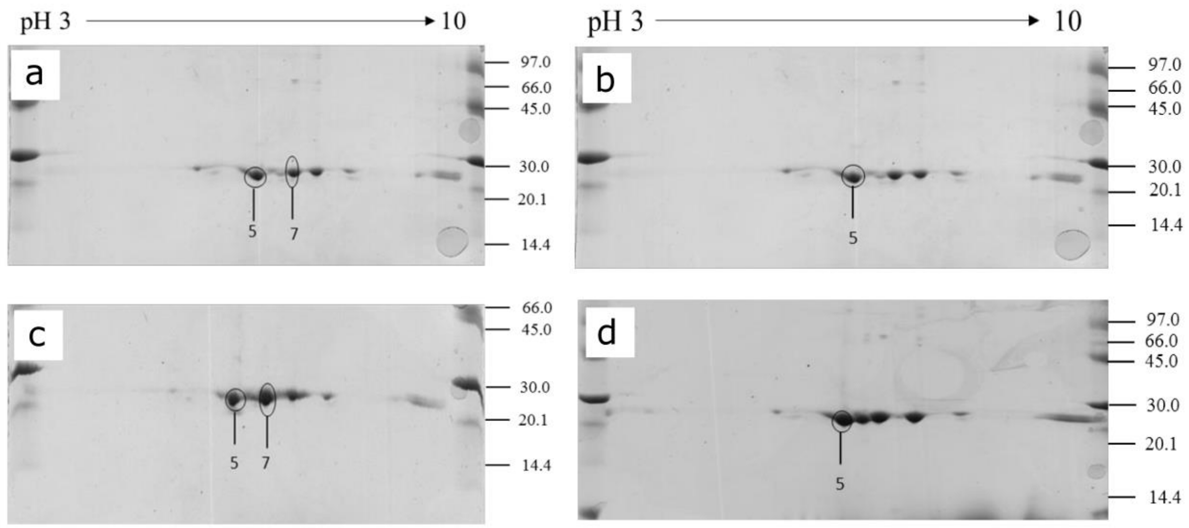

Online Resource 3 Comparison of the 2-DE arrays for each TCBZ-SO treatment group via Progenesis. Comparison of the average Control (A) vs. average Sub-lethal treatment (B) purified via GSH agarose identified an increase in normalised spot volumes for two spots, spots 5 and 7. Comparison of the average Control (C) vs. average Lethal (D) purified via GSH agarose identified an increase in normalised spot volumes for spot 5. Proteins were separated across a linear pH range 3 -10 using IEF in the first dimension and 12.5% SDS-PAGE in the second dimension and Coomassie Blue-stained
