# Supplementary material for: The soluble glutathione transferase superfamily: role of Mu class in triclabendazole sulphoxide challenge in Fasciola hepatica
Source: Parasitol Res. 2021 Jan 27;120(3):979–91. doi: 10.1007/s00436-021-07055-5 (PMC7889535; doi:10.1007/s00436-021-07055-5)
Supplement: Supplementary file 4 — (PDF 185 kb) [file 436_2021_7055_MOESM4_ESM.pdf]

Online Resource 4 Transcript support for the newly identified FhGST-S2 and FhGST-O1. BLAST hits following analysis of databases using FhGST-S1, -S2, -O1 and -O2 are provided. Top scoring hits are shaded in grey. Transcripts were retrieved from [A]Young *et al*. (2011), [B]Young *et al*. (2010), [C]an in house *F. gigantica* newly excysted juvenile transcriptome, [D]the available EBI-ENA archive ERP000012: an initial characterization of the *F. hepatica* transcriptome using 454-FLX sequencing and [E]from *F. hepatica* ESTs available by anonymous FTP from the Wellcome Trust Sanger Institute ftp://ftp.sanger.ac.uk/pub/pathogens/Fasciola/

**Sigma Class GSTs**	**GST-S1 Scaffold1081**	**GST-S2 Scaffold1104**
Contig24647[A]	1.00E-33	6.00E-17
Contig27045[A]	2.00E-10	-
Fh_Contig8894[B]	4.00E-50	5.00E-24
isotig05100[C]	8.00E-50	2.00E-23
isotig05101[C]	8.00E-50	2.00E-23
isotig04409[C]	6.00E-14	5.00E-50
contig07874[D]	6.00E-50	1.00E-23
contig06001[D]	7.00E-14	6.00E-50
**Omega Class GSTs**	**GST-O1 Scaffold1029**	**GST-O2 Scaffold50**
contig03575[D]	3.00E-11	2.00E-45
contig04426[D]	2.00E-45	2.00E-11
HAN5016c12.q1kT3[E]	5.00E-04	5.00E-24
isotig09504[C]	1.00E-10	3.00E-44
isotig10825[C]	1.00E-42	9.00E-12
Contig20835[A]	1.00E-10	3.00E-44
Contig13488[A]	5.00E-35	4.00E-09
Contig12260[A]	3.00E-43	2.00E-12
Fh_Contig5261[B]	2.00E-45	2.00E-11
Fh_Contig2859[B]	6.00E-11	1.00E-44
