# Supplementary material for: The soluble glutathione transferase superfamily: role of Mu class in triclabendazole sulphoxide challenge in Fasciola hepatica
Source: Parasitol Res. 2021 Jan 27;120(3):979–91. doi: 10.1007/s00436-021-07055-5 (PMC7889535; doi:10.1007/s00436-021-07055-5)
Supplement: Supplementary file 5 — (PDF 1015 kb) [file 436_2021_7055_MOESM5_ESM.pdf]

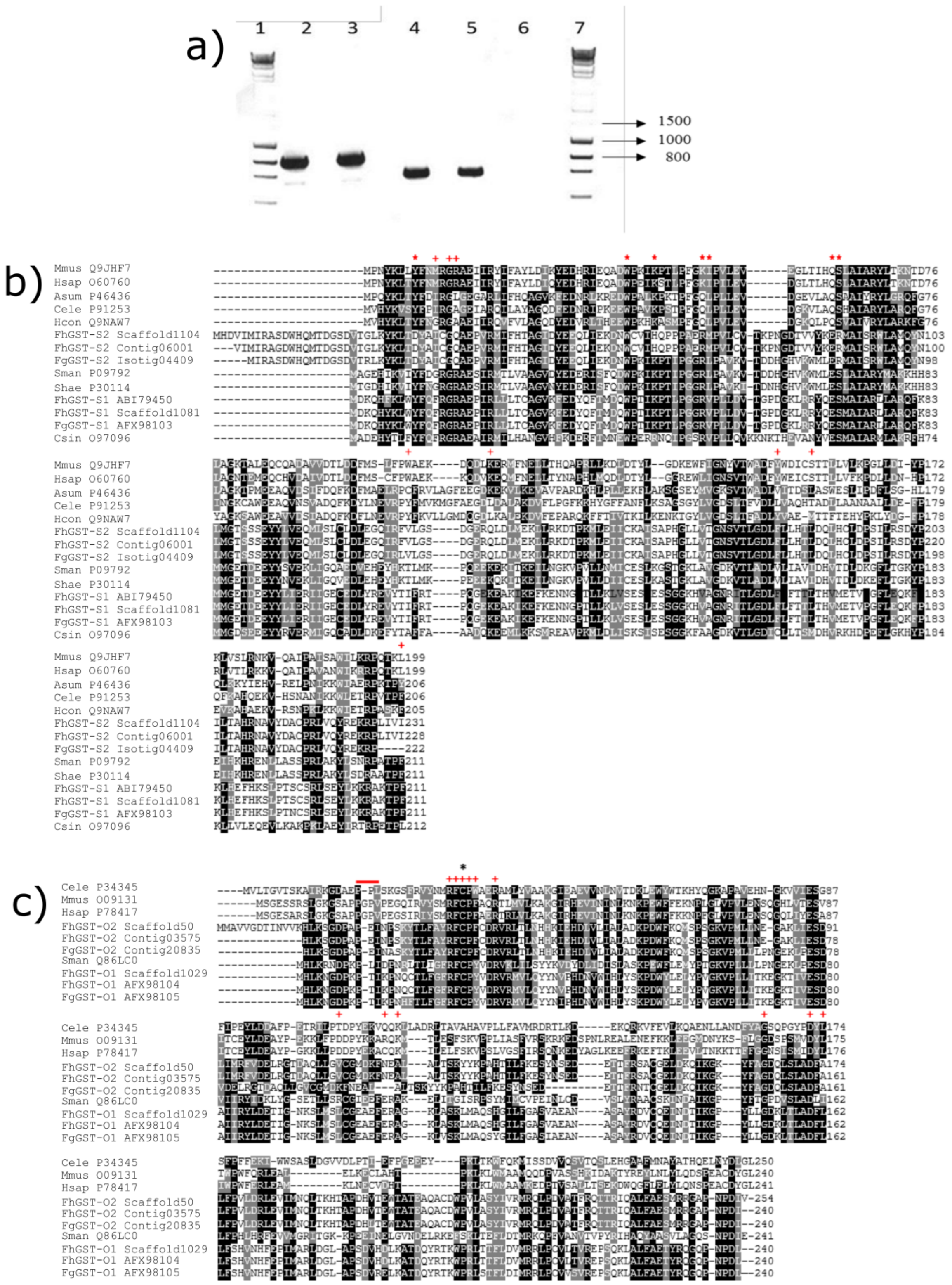

Online Resource 5 Bioinformatics and cloning of the newly identified FhGST-S2 and FhGST-O2. A) PCR amplification of FhGST-O2 and FhGST-S2: 1% agarose gel displaying full PCR products of FhGST-O2 (Lane 2 and 3) and FhGST-S2 (lane 4 & 5). Negative control in lane 6. Replicate lanes represent different individual cDNAs. Both products, for FhGST-O2 and FhGST-S2, were subsequently cloned into pGEM-T-Easy and sequenced in

house. B) Multiple alignment of *Fasciola* Sigma class GST protein sequences with those of genome sequenced organisms. All GST sequences were aligned using Clustal W. The amino acids involved in the formation of glutathione-binding and catalytic sites are marked with a plus (+) and asterisks (*), respectively. C) Multiple alignment of *Fasciola* Omega class GST protein sequences with those of genome sequenced organisms. All GST sequences were aligned using Clustal W. Indicated by a solid line are the proline-rich residues in the Omega class characteristic N-terminal extension. The catalytic cysteine residue characteristic of Omega class GSTs is indicated with *. Residues indicated with + relate to the identified Omega class GST motifs identified by Chemale *et al*. (2006). Species abbreviations used are Mmus, *Mus musculus*; Hsap, *Homo sapiens*; Asum, *Ascaris suum*; Cele, *Caenorhabditis elegans*; Hcon, *Haemonchus contortus*; Sman, *Schistosoma mansoni*; Shae, *Schistosoma haematobium*; Csin, *Clonorchis sinensis*
