# Supplementary material for: The soluble glutathione transferase superfamily: role of Mu class in triclabendazole sulphoxide challenge in Fasciola hepatica
Source: Parasitol Res. 2021 Jan 27;120(3):979–91. doi: 10.1007/s00436-021-07055-5 (PMC7889535; doi:10.1007/s00436-021-07055-5)
Supplement: Supplementary file 6 — (PDF 95 kb) [file 436_2021_7055_MOESM6_ESM.pdf]

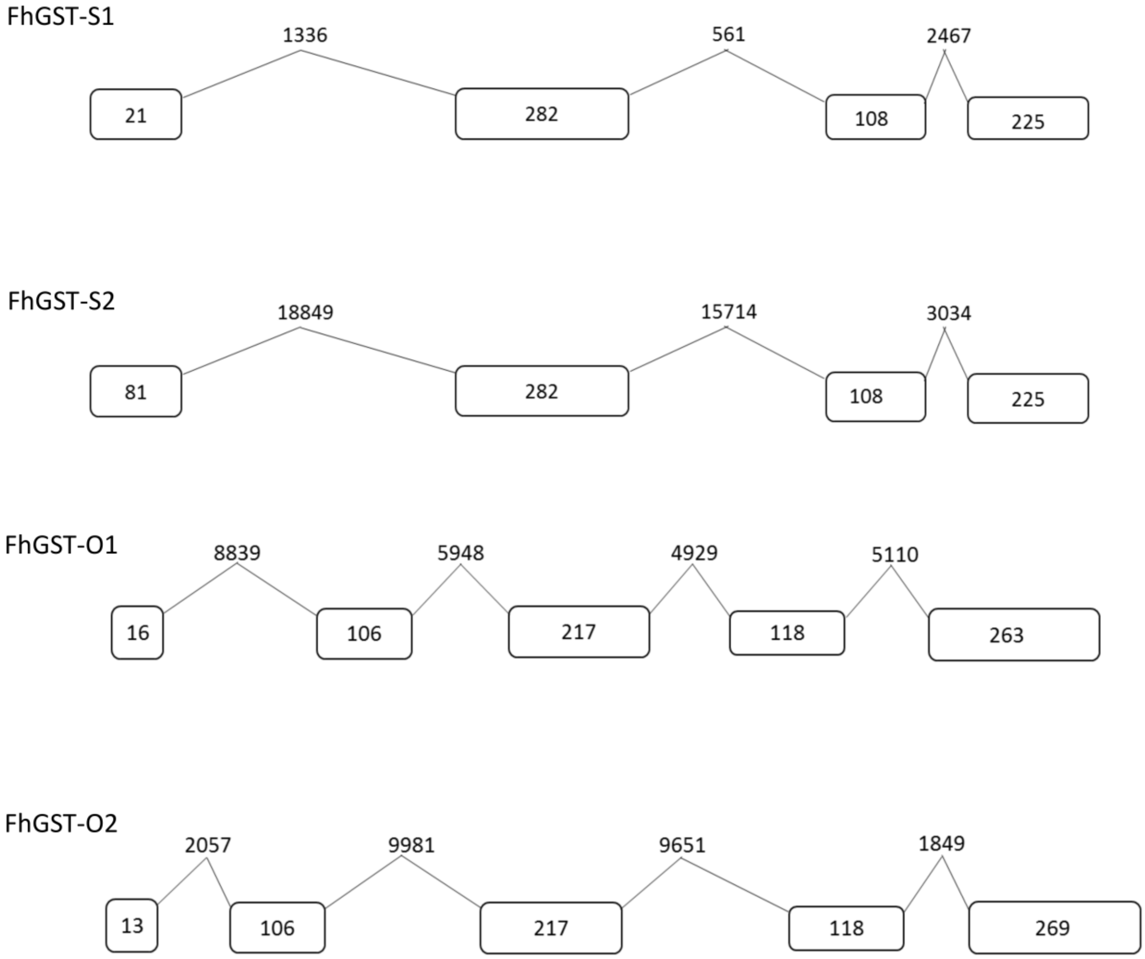

Online Resource 6 Comparison of exon-intron architectures of sigma and omega class GST genes from *F. hepatica*. Protein-coding regions are shown as boxes, and intervening introns are demonstrated by solid thin lines. The lengths of exons and introns in base pairs are presented at the corresponding positions. Of note is the N terminal extension in exon 1 of FhGST-S2. FhGST-S1 (Scaffold 1081), FhGST-S2 (Scaffold 1104), FhGST-O1 (Scaffold 1029) and FHGST-O2 (Scaffold 50)
